# The Effects of a Microbial Enzyme Mixture on Macronutrient Hydrolysis in a Static Simulation of Oro-Gastric Digestion That Models Human Digestive Senescence

**DOI:** 10.3390/foods14060937

**Published:** 2025-03-10

**Authors:** Sean M. Garvey, Erin N. Madden, Yunyao Qu, Caroline H. Best, Kelly M. Tinker

**Affiliations:** Department of Research and Development, BIO-CAT, Inc., Troy, VA 22974, USA

**Keywords:** aging, amylase, dietary protein, digestive enzyme, INFOGEST, lipase, protease

## Abstract

Observational studies have shown that human digestive function declines naturally with age. Oral enzyme supplementation is a candidate strategy to enhance macronutrient digestion in older adults. The objective of this study was to test the effects of a mixture of six microbial enzyme preparations (ENZ) on nutrient bioaccessibility from a mixed meal in an in vitro model of digestive senescence. The mixed meal included chicken meat, peas, and potatoes. The INFOGEST 2.0 static simulation of oro-gastric digestion was used to model human digestive physiology along with a consensus protocol to model aging. Analytical testing of gastric digesta included measurements of free amino nitrogen (FAN), amino acid (AA), fatty acid (FA), glycerol, maltose, and glucose concentrations. Peptide distribution profiles were evaluated by size exclusion chromatography (SEC) and gel electrophoresis. After simulating digestion of the mixed meal, all nutrient bioaccessibility outcomes compared to pepsin-only controls, except glycerol, were further enhanced by ENZ in the aging condition compared to the standard condition (FAN: 77.1 vs. 39.3%; essential AA: 100.4 vs. 57.6%; total FA: 12.8- vs. 8.0-fold; maltose: 142.1 vs. 0.7%). SEC confirmed ENZ’s proteolytic capacity to generate more lower molecular weight peptides and free AAs in standard and aging conditions compared to pepsin alone. Gel electrophoresis confirmed proteolytic enhancement with ENZ. These data showcase ENZ’s hydrolytic activity toward macronutrients and suggest ENZ’s capacity to compensate for reduced pepsin activity in an aging-adapted oro-gastric digestion simulation.

## 1. Introduction

Clinical observational studies of digestive function in younger and older adults have shown that digestive function begins to decline as early as the fifth decade of life [1]. For example, at least three studies have concluded that gastric pepsin output declines by up to 40% with advancing age [2,3,4]. Reduced gastric acidity (i.e., hypochlorhydria) has also been observed in older adults compared to younger adults [5], as well as delayed postprandial return to typical fasting gastric acidity [6]. These age-associated changes could theoretically limit pepsin- and acid-mediated protein digestion in the stomach. Another age-related change in gastric physiology is slower gastric emptying [7,8,9,10]. Observational studies have also suggested that pancreatic enzymes show decreased output or activity with older age [4,11,12,13,14]. Three of these studies showed evidence for decreased pancreatic bicarbonate secretion with age [12,13,14], which may lead to decreased duodenal pH and pancreatic enzyme activity [15]. Intestinal bile concentration has also been noted to decline with age [16,17], potentially lessening dietary fat emulsification, lipase-mediated triglyceride hydrolysis, and the bioaccessibility of fatty acids and sn-2-monoacylglyercols. Despite this body of evidence supporting age-associated decline in digestive function, Sanders et al. [18] cited other literature—beyond the scope of this article—and concluded that the digestive system is resilient and sufficiently functional with advancing age in healthy adults.

Nonetheless, the aforementioned observational studies highlight a potential phenomenon which may be referred to as digestive senescence or age-related digestive resistance. Related observational studies have convincingly shown that appetite and food intake decline with increasing age [19], reflecting a condition referred to as “anorexia of the elderly” by Morley et al. [20]. First and foremost, older adults at risk for malnutrition, weight loss, and sarcopenia stand to benefit from dietetic counseling and nutritional therapies [21,22,23]. Additionally, exogenous digestive enzyme supplementation remains a candidate approach to compensate for digestive senescence in older adults who are at greater risk for undernutrition and malabsorption. Supplemental enzymes may particularly be helpful for older adults who (i) have a reduced appetite and eat less (i.e., the anorexia of aging), (ii) have concurrent gastrointestinal (GI) disorders (e.g., gastritis or achlorhydria), or (iii) take certain medications with side effects that reduce nutrient bioaccessibility or absorption (e.g., proton pump inhibitors, histamine type 2 receptor antagonists, antacids, or certain antibiotics like tetracycline).

Compared to supplemental porcine enzymes like pepsin and pancreatin, enzymes obtained by microbial fermentation (i.e., “microbial enzymes”) often carry the advantage of tolerance to and activity in conditions that simulate typical human gastric acidity and pepsin exposure in vitro [24,25,26,27]. Microbial enzyme activity has also been demonstrated in the GI tract of experimental mice following oral administration [28,29]. Microbial enzymes are routinely used as feed additives to support gut health and improve feed conversion efficiency in livestock and aquaculture [30,31]. In humans, the oral administration of microbial enzymes has been shown to reduce the severity of GI symptoms in individuals with functional dyspepsia or non-celiac gluten sensitivity [32,33], reduce abdominal distention in healthy adults [34], and improve protein digestion in healthy adults [35,36]. However, very little is known about supplemental enzyme benefits in older adults.

Prior to clinical investigation, it is critical to establish enzyme efficacy and dosing in physiologically relevant in vitro digestion models. The 2011 European Cooperation on Science and Technology Action No. FA1005 titled “Improving health properties of food by sharing our knowledge on the digestive process” (also known as INFOGEST) resulted in a global network of scientists and the publication of a consensus protocol for static GI digestion simulation in 2014 [37]. An upgraded static INFOGEST protocol, version 2.0, was published in 2019 and now has over 2600 citations [38]. Static protocols enable rapid experimentation since pH adjustments, digestive fluid volumes, and enzyme inputs occur once at the beginning of the oral, gastric, and intestinal phases. This approach contrasts dynamic and semi-dynamic protocols with instrumentation that constantly or periodically adjusts the pH, digesta flux, and enzyme inputs to more precisely model human digestive physiology [39]. However, the static INFOGEST simulation has been shown to be physiologically comparable to in vivo pig digestion [40].

Using the static INFOGEST 2.0 simulation of GI digestion, dose–response experiments were performed to help define a precise mixture of six microbial enzyme preparations (hereafter referred to as “ENZ”) that enhanced amino acid, glycerol, maltose, and glucose bioaccessibility from a mixed meal [24]. In that study, ENZ also improved nutrient bioaccessibility in conditions that modeled aging rather crudely by the use of 30% less pepsin in the gastric phase compared to the standard consensus protocol [24]. To complement that work, the aim of this research was to test the effects of ENZ on macronutrient digestion in the 2023 consensus static INFOGEST protocol adapted to model aging [41]. The key changes in this aging-adapted protocol for oro-gastric digestion simulations reflect the aforementioned human physiological observations and include (i) increasing the starting gastric pH from 3.0 to 3.7, (ii) reducing the pepsin concentration in the gastric phase by 40%, and (iii) extending the duration of the gastric phase from 2 h to 3 h [41]. All oro-gastric simulations were conducted with a mixed meal substrate comprising chicken meat, sweet peas, instant potatoes, butter, and milk. To assess nutrient bioaccessibility, gastric digesta free amino nitrogen (FAN), amino acid, fatty acid, glycerol, maltose, and glucose concentrations were measured. To extend prior work, proteolysis was also determined through an evaluation of peptide distribution by size exclusion chromatography and gel electrophoresis. ENZ was predicted to improve nutrient bioaccessibility compared to pepsin alone in the standard and aging conditions.

## 2. Materials and Methods

### 2.1. Enzymes

The supplemental mixture of six non-genetically engineered microbial enzyme preparations (Trade Name: OPTIZIOME^®^ Macro Digest, Lot No. OZMD-ZH30, BIO-CAT, Inc., Troy, VA, USA), hereafter referred to as “ENZ”, is described in Table 1. The ENZ mixture was formulated to contain each of the enzyme preparations and activities listed in Table 1 based on measurements of enzyme activities using separate assays specific to each of the enzyme preparations, as previously detailed in [24]. Of note, the three protease preparations were manufactured by traditional Japanese kōji fermentation of wild-type *Aspergillus* species. Pepsin from porcine gastric mucosa was purchased from MilliporeSigma^®^ (Product No. P6887, MilliporeSigma, Burlington, MA, USA). Details regarding the assay used to measure porcine pepsin activity are provided elsewhere [24,38].

### 2.2. Substrate

The mixed meal for digestion simulations included grilled chicken breast meat (Perdue^®^ SHORT CUTS^®^ Grilled Chicken Strips, Perdue Foods LLC, Salisbury, MD, USA), steamed sweet peas (Birds Eye^®^ Steamfresh^®^ Sweet Peas, Pinnacle Foods Group LLC, Parsippany, NJ, USA), instant mashed potatoes (Idahoan^®^ Original Mashed Potatoes, Idahoan Foods, LLC, Idaho Falls, ID, USA), unsalted butter (Great Value^®^, Walmart Apollo, LLC, Bentonville, AK, USA), and bovine milk (PET^®^ 2% Reduced Fat Milk, Eagle Family Foods Group LLC, Richfield, OH, USA). To prepare the mixed meal, the frozen chicken strips (previously fully cooked and then frozen by the manufacturer) and peas were heated in a microwave for 90 s according to the manufacturer’s directions. To simulate human mastication, peas and chicken meat were minced to a particle size of 2–3 mm with a mixer (Model No. KSM75WH, KitchenAid^®^ Classic Plus, KitchenAid, Benton Harbor, MI, USA) and grinder attachment (KITOART^®^ Food Grinder Attachment, Model No. KTA G1, available through Amazon.com, Inc.). Peas were minced with a coarse plate and a 4-sided blade, while the chicken meat was minced with the medium plate and a 2-sided blade. To prepare the mashed potatoes, 237 mL of boiling water was added to 65 g of potato flakes along with 118 mL of milk and stirred vigorously for 90 s. A partial 1/40th serving of each mixed meal component ingredient (chicken: 1.8 g; peas: 2.9 g; mashed potatoes: 4.8 g; and melted butter: 0.5 g) was weighed separately and transferred to 250 mL round plastic jars (Nalgene^®^ Straight-Sided Polypropylene Jars, Nalge Nunc Corporation, Rochester, NY, USA). Note that one full serving of the mixed meal contains 34 g of protein, 21 g of fat, and 51 g of carbohydrates per serving, as previously described [24]. The mixed meal was designed to (i) result in a proportion of macronutrients that align with the daily acceptable macronutrient distribution range proposed by the Institute of Medicine [42], (ii) include an adequate amount of protein based on acute feeding studies to support muscle metabolism in aging adults [43], (iii) contain a source of dietary fiber that is not overly lignocellulosic (i.e., peas) in order to promote digesta homogeneity and experimental reproducibility in a partial serving digestion simulation, (iv) reflect a typical meal consumed and manufactured from ingredients harvested or grown in the United States (i.e., one meat, one vegetable, and one predominant source of starch), and (v) include ingredients that could easily be purchased by other laboratories, including an iteration of this meal using canned foods [24].

### 2.3. In Vitro Static Simulation of Oro-Gastric Digestion

The static INFOGEST simulation of GI digestion has previously been described [37,38]. To prepare the oral phase, 10 g of minced mixed meal was transferred to 250 mL Nalgene^®^ round plastic jars. The jars were placed in a 20 L PolyScience^®^ water bath (Model No. WBE20A11B, Preston Industries Inc., Niles, IL, USA) on a submersible stir plate (Cimerec™ iTelesystem 6 Multipoint Stirrers, Thermo Fisher Scientific Inc., Waltham, MA, USA) at 37 °C. Simulated salivary fluid (10 mL) was added to the mixed meal (1:1 vol-to-wt ratio) through magnetic stirring for 2 min at 200 rpm (amylase was not included). To initiate the 2 h gastric phase, simulated gastric fluid (19 mL) with porcine pepsin (1 mL) was added to the oral digesta (1:1 vol-to-vol ratio) and adjusted to either pH 3.00 ± 0.02 for the standard condition (i.e., young to middle-aged adult condition) or pH 3.70 ± 0.02 for the aging condition, using 1N HCl or 1N NaOH. Standard gastric phase conditions included a starting gastric pH of 3, pepsin concentration of 2000 U/mL, and a 2 h duration. Gastric phase conditions modeling advanced age included a starting gastric pH of 3.7, pepsin concentration of 1200 U/mL, and a 3 h duration [41]. Simulations were performed with and without the addition of ENZ 10 min into the gastric phase to model the approximate dissolution time of a cellulose-based capsule shell at typical gastric pH [44]. In each individual experiment, ENZ was investigated at a fractional 1/40th dose, commensurate with the 1/40th partial serving size (10 g) of the mixed meal. At the conclusion of the 2 or 3 h gastric phase for standard or aging conditions, respectively, gastric digesta samples were transferred to 50 mL conical tubes and placed in a 90 °C water bath for 10 min to inactivate enzymes. After cooling to room temperature, samples were refrigerated at 4 °C or frozen at −20 °C before analytical testing.

### 2.4. Free Amino Nitrogen Analysis

The extent of protein hydrolysis after digestion simulations was investigated by the measurement of free amino nitrogen (FAN) in gastric digesta samples using the alpha-amino nitrogen by o-phthaldaldehyde (NOPA) method [45]. Two reagents and standards were prepared. Reagent “A” was prepared by dissolving 0.3837 g of sodium hydroxide, 0.8468 g of boric acid, and 0.0816 g of N-acetyl-L-cysteine (NAC) in 75 mL of deionized water on a magnetic stir plate. Separately, 0.0671 g of o-phthaldaldehyde (OPA) was dissolved in 10 mL of 95% ethanol and transferred to the NaOH/boric acid/NAC solution by rinsing with deionized water. This mixture was transferred to a volumetric flask and diluted to 100 mL with deionized water. To prepare Reagent “B”, 0.3837 g of sodium hydroxide, 0.8468 g of boric acid, and 0.0816 g of NAC were dissolved in 75 mL of deionized water on a magnetic stir plate, to which 10 mL of 95% ethanol was added and diluted to 100 mL in a volumetric flask with deionized water. Isoleucine standards were prepared by first dissolving 0.328 g of L-isoleucine (Product No. I2752, MilliporeSigma) in deionized water in a 250 mL volumetric flask. In a 96-microwell plate, 300 µL of Reagent A was added to 5 µL of each standard dilution and mixed. After 10 min, absorbance was determined at 335 nm using a SpectraMax^®^ PLUS 384 Microplate Reader (Molecular Devices^®^, LLC, San Jose, CA, USA), and a calibration curve was created by water subtraction. For experimental samples, 300 µL of Reagent A was added to 5 µL of each gastric digesta sample, mixed, incubated for 10 min, and analyzed at 335 nm. For the blanks, 300 µL of Reagent B was added to 5 µL of each gastric digesta sample, mixed, incubated for 10 min, and analyzed at 335 nm, and this value was subtracted from the corresponding sample measurement with Reagent A to calculate net absorbance. The FAN release is calculated using the following equation:

mg FAN/g = (net absorbance × slope) × dilution factor/1000

Results are reported in mg free alpha-amino nitrogen per g of mixed meal.

### 2.5. Amino Acid Analysis

The concentrations of the 20 amino acids (AAs) comprising dietary protein were measured in gastric digesta by fluorescence detection using an Agilent^®^ 1200 Series HPLC (Agilent Technologies, Inc., Santa Clara, CA, USA). Standards (Amino Acid Standard H, Reference No. 20088, Thermo Fisher Scientific Inc.; Amino 254 Acid Supplement Kit, Part No. 5062-2478, Agilent Technologies, Inc.) and samples were prepared in 0.1 N HCl. All gastric digesta samples were thawed, vortexed, and allowed to sediment before aliquots of supernatants were diluted with 0.1 N HCl and filtered through 0.2 μm polyvinylidenedifluoride (PVDF) filters (Part No. 5190-5263, Agilent Technologies, Inc.). Samples (5 µL) were separated on a ZORBAX^®^ Eclipse Plus C18 column (4.6 × 150 mm, Part No. 959993-902, Agilent Technologies, Inc.) at 40 °C with a flow rate of 1.5 mL/min. Gradient elution started with a mixture of 10 mM sodium phosphate, 10 mM sodium tetraborate, and 5 mM sodium azide at 98% vol/vol (pH 8.2), with the remaining 2% vol/vol comprising acetonitrile, methanol, and water (45:45:10 vol ratio), and ended with 100% acetonitrile, methanol, and water (45:45:10 vol ratio). Samples were first buffered with 0.4 M borate buffer at pH 10.2. Primary AAs were derivatized with OPA in the presence of 3-mercaptopropionic acid. Secondary AAs were derivatized using 9-fluorenylmethyl chloroformate. Results are reported in mg AA per g of mixed meal.

### 2.6. Size Exclusion Chromatography

The peptide size distributions of gastric digesta were analyzed by size exclusion chromatography (SEC) with UV detection at 214 nm using an Agilent^®^ 1100 Series HPLC according to previously published methods [46]. Briefly, peptide size standards were prepared at 2 mg/mL in deionized water (i.e., tryptophan (molecular weight (MW)—204 Da; retention time (RT)—10.98 min); leucine enkephalin (MW—570 Da; RT—10.44 min); angiotensin II (MW—1046 Da; RT—8.15 min); insulin chain B (MW—3496 Da; RT—7.82 min); aprotinin (MW—6511 Da; RT—6.54 min); lysozyme (MW—14,300 Da; RT—6.24 min); carbonic anhydrase (MW—29,000 Da; RT—5.96 min); ovalbumin (MW—42,700 Da; RT—5.93 min)). Gastric digesta samples were thawed, vortexed, and allowed to sediment before aliquots of supernatant were diluted with deionized water and filtered through 0.2 μm PVDF filters. Filtrates (10 µL) were separated on a BioSep™ SEC-s2000 column (7.8 × 300 mm, Cat No. 00H-2145-K0, Phenomenex^®^, Inc., Torrence, CA, USA). The eluent consisted of a mixture of acetonitrile (30% vol/vol) and deionized water (70% vol/vol) with 0.05% trifluoroacetic acid with a flow rate of 0.9 mL/min. The MWs of the proteins were determined by plotting the RTs of the standards against their corresponding MWs to generate a calibration curve.

### 2.7. Gel Electrophoresis

Preparation and analysis of gastric digesta samples using sodium dodecyl-sulfate polyacrylamide gel electrophoresis has previously been described [47]. Briefly, samples were diluted to 30 mg total protein/mL, based on Lowry assay, and 10 µL was loaded on the gel. Technical duplicates of a single experiment are shown (*n* = 1).

### 2.8. Glycerol, Maltose, and Glucose Analyses

Glycerol, maltose, and glucose were measured using an Agilent^®^ 1100 Series HPLC with refractive index detection. Standards were purchased from MilliporeSigma and included glycerol (Product No. G7893, Lot No. SHBM1148), maltose (Product No. M5885, Lot No. SLBN0860V), and glucose (Product No. G5767, Lot No. SLBP5997V). Gastric digesta samples were thawed and passed through 0.45 µm nylon syringe filters (Part No. CH4525-NN; Thermo Fisher Scientific Inc.), and 10 µL filtrate was injected and separated on a Supelco™ SUPELCOGEL Ca 7.8 × 300 mm column (Cat No. 59305-U; MilliporeSigma) at a flow rate of 0.5 mL/min with deionized water as the eluent. Glycerol concentrations of digesta samples were below the limit of quantification and close to the limit of detection. In order to quantify glycerol, an amount of glycerol standard equivalent to 0.1 mg/mL was added to each filtered sample. This standard amount was then subtracted from the final value. Calibration curves for standards were generated at 0.05, 0.1, 0.25, 0.5, 0.75, and 1 mg/mL concentrations. Results are reported in mg analyte per g of mixed meal.

### 2.9. Fatty Acid Analysis

Fatty acid (FA) concentrations of gastric digesta were measured using an Agilent^®^ 1100 Series HPLC with UV detection at 208 nm similar to previously published methods [48]. Standards included lauric acid (C12:0, Product No. L0011, TCI AMERICA, Portland, OR, USA), myristic acid (C14:0, Product No. M0476, TCI AMERICA), oleic acid (C18:1n9, Product No. O1008, MilliporeSigma), and palmitic acid (C16:0, Product No. P0500, MilliporeSigma). Gastric digesta samples were thawed and centrifuged at 2750 × *g* for 10 min. Due to the low overall concentration of lipids in the digesta, just the top oil layer was collected for FA analysis and diluted in acetone. Samples (20 µL each) were separated on a Supelco^®^ Discovery^®^ HS C18 column (4.6 × 250 mm, Cat No. 568523-U; MilliporeSigma). A mixture of acetonitrile (90% vol/vol), methanol (8% vol/vol), and hexane (2% vol/vol) with 0.2% acetic acid was used as eluent with a flow rate of 1.0 mL/min. Calibration curves for lauric acid, myristic acid, oleic acid, and palmitic acid were constructed since these were the four most predominant FAs detected and are known to be abundant in bovine milk and butter [49]. Total FA in the context of this work reflects the sum of these four aforementioned FAs. Results are reported in mg of total FA per g of oil in a layer of the mixed meal.

### 2.10. Statistics

Statistical analyses were performed using GraphPad^®^ Prism^®^ software (version 10.2.3, GraphPad Software, Inc., San Diego, CA, USA). A two-way analysis of variance (ANOVA) was conducted to evaluate the effects of treatment (control or ENZ use) and INFOGEST simulation condition (standard or aging), as well as their interaction, on the FAN, total AA, total essential amino acid (EAA), total branched chain amino acid (BCAA), leucine, glycerol, total FA, maltose, and glucose concentrations of gastric digesta. Assumptions of normality and homoscedasticity were assessed using QQ plots, residual plots, and homoscedasticity plots, with no violations detected. Following ANOVA, uncorrected Fisher’s least significant difference test was applied for pairwise comparisons within each INFOGEST treatment and condition group.

## 3. Results and Discussion

In vitro oro-gastric digestion simulations of a mixed meal were carried out under standard or aging conditions and with pepsin alone (control) or pepsin with a mixture of six microbial enzyme preparations (ENZ). Descriptive and inferential statistics for end-of-simulation gastric digesta free amino nitrogen (FAN), total amino acid (AA), total essential amino acid (EAA), total branched chain amino acid (BCAA), leucine, glycerol, total fatty acid (FA), maltose, and glucose concentrations are provided in Table 2.

### 3.1. pH Response Profiling

**Figure 1:**
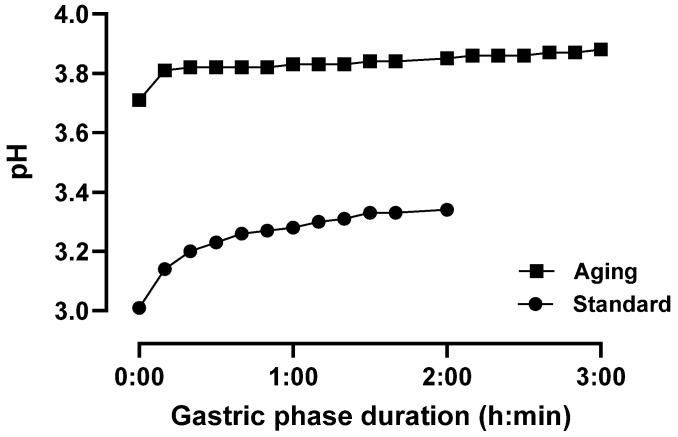
The evolution of gastric phase pH during the simulated oro-gastric digestion of a mixed meal under standard INFOGEST 2.0 conditions (Standard) or conditions that model digestive decline with advancing age (Aging). Longitudinal pH measurements were carried out with digesta samples from a single simulation under each condition (*n* = 1).

Because the aging condition includes a starting gastric pH of 3.7 compared to pH 3.0 in the standard condition, the evolution of pH during the gastric digestion simulation using the mixed meal substrate was first evaluated. In the standard condition with pepsin alone, the pH ranged from 3.01 to 3.34 during the 2 h gastric phase (Figure 1). In the aging condition, the pH ranged from 3.71 to 3.88 during the 3 h gastric phase (Figure 1). This food buffering effect on gastric pH is well established and tends to be greater with high-protein foods [50,51], such as the mixed meal studied here.

### 3.2. Peptide and Amino Acid Bioaccessibility

#### 3.2.1. Free Amino Nitrogen

**Figure 2:**
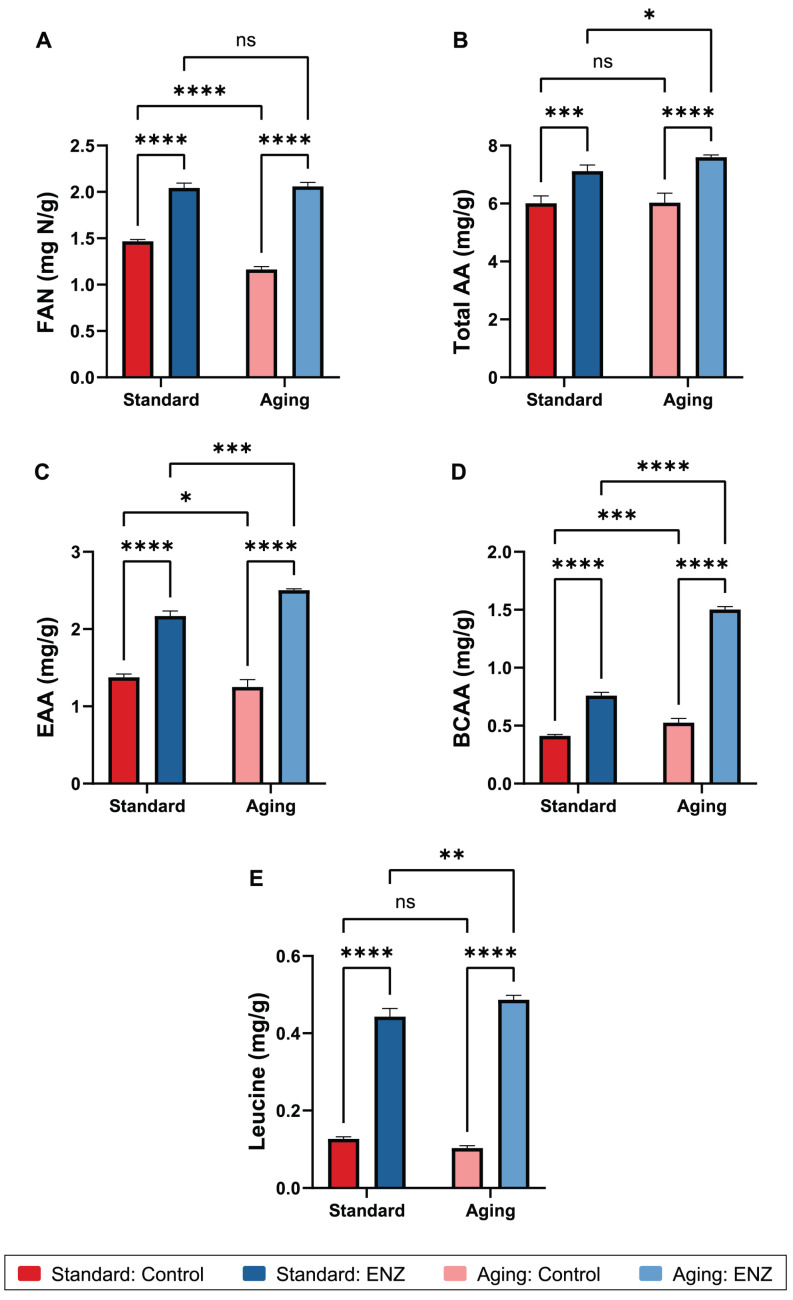
The average gastric digesta free amino nitrogen (FAN) (**A**), total amino acid (AA) (**B**), total essential amino acid (EAA) (**C**), total branched chain amino acid (BCAA) (**D**), and leucine (**E**) concentrations after oro-gastric simulations of mixed meal digestion with and without ENZ under standard INFOGEST 2.0 conditions (Standard) or conditions that model digestive decline with advancing age (Aging). The control simulations included porcine pepsin in the gastric phase. The ENZ simulations included porcine pepsin in the gastric phase along with a mixture of six microbial enzyme preparations. The results are reported in mg of N or mg of AA per g of mixed meal. The error bars show ± one standard deviation (*n* = 3 per group). *, *p* ≤ 0.05; **, *p* ≤ 0.01; ***, *p* ≤ 0.001; ****, *p* ≤ 0.0001; ns, not significant (*p* > 0.05).

In a comparison of just the control simulations with pepsin alone (i.e., no ENZ included), the average FAN concentration of gastric digesta was reduced by 20.7% in the aging condition compared to the standard condition (1.16 vs. 1.47 mg N/g, *p* = 0.0002, Figure 2A and Table 2). The extended duration of the gastric phase in the aging condition from the standard 2 h duration to 3 h did not compensate for the reduced pepsin concentration (Figure 2A). This decrease in peptic N-termini generation at reduced gastric acidity in the aging vs. standard condition (pH 3.7 vs. pH 3.0) is expected since 40% less pepsin is used in the aging condition. Historically, pepsin’s primary endopeptidase activity was described as optimal at up to pH 4 and prone to decrease steeply beyond pH 4 [52]. It is now understood that pepsin’s pH response is substrate-dependent with less loss of proteolytic activity between pH 3 and 4 for certain substrates [53,54]. A preliminary analysis of pepsin activity across a range of starting gastric pH values in the standard INFOGEST 2.0 static condition shows comparable FAN and AA release between pH 3 and 4.5 (Figure S2), suggesting that it is reduced pepsin usage and neither reduced pepsin activity (per unit pepsin) nor reduced acid hydrolysis that yields less proteolysis of the mixed meal in the aging condition. In contrast, reduced pepsin activity and acid hydrolysis were considered to contribute to the decreased release of FAN from a mixed meal in a semi-dynamic INFOGEST gastric simulation adapted to model proton pump inhibitor use, whereby the target final gastric pH was set to 4.2 compared to pH 2.0 in the standard condition [55].

Reduced dietary protein hydrolysis in the consensus INFOGEST aging condition, compared to the standard condition, has previously been observed in simulations of oro-gastric or gastric digestion of lentil and quinoa seed flours [56], high-protein dairy products [57,58], bovine α-lactalbumin [59], cooked pork [60], a panel of raw tilapia meat, raw chicken breast meat, raw crab meat, soybean protein isolate [61], and cold cut turkey meat or cheese [62]. In the case of pasta, though, the extended duration of the gastric phase in another study appears to have improved protein digestion in the aging condition compared to the standard condition, perhaps owing to the highly processed nature of the protein source [63].

The impact of ENZ use in the gastric phase was also evaluated in both conditions. In the standard condition, ENZ increased the average digesta FAN concentration by 39.3% compared to the control (2.04 vs. 1.47 mg N/g, *p* < 0.0001; Figure 2A). In the aging condition, ENZ increased the average FAN digesta concentration by 77.1% compared to the control (2.06 vs. 1.16 mg N/g, *p* < 0.0001; Figure 2A). It is noteworthy that ENZ promoted a nearly identical absolute release of FAN under both standard and aging conditions (2.04 vs. 2.06 mg N/g, *p* = 0.6095; Figure 2A), suggesting that ENZ’s proteolytic activity is effective at a gastric pH of 3.7 and higher and compensates for a diminished pepsin concentration in the aging condition. Moreover, a significant interaction between ENZ use and condition was observed (*p* < 0.0001), indicating that the relative increase in FAN with ENZ was more pronounced in the aging condition compared to the standard condition. The hemoglobin unit tyrosine base (HUT) assay modified to evaluate activity across pH 2 to 6 was initially suggestive of higher ENZ proteolytic activity at pH 3.7 and pH 4.0 compared to pH 3.0 and below (Figure S1). Because the HUT assay is specific to tyrosine release from bovine hemoglobin, pH response profiling was also performed in the INFOGEST simulation. A preliminary analysis of ENZ across a range of starting gastric pH from 3.0 to 4.5 in the standard condition showed that pH had little effect on FAN release from the mixed meal (Figure S2). These data corroborate the known optimal pH ranges of the two most abundant proteases in the ENZ mixture—*Aspergillus oryzae* protease and *A. niger* protease (Table 1). Thus, ENZ-mediated proteolysis in the aging condition is likely driven by the extended duration of the gastric phase; however, digesta analyses at intermediate timepoints are needed to confirm this.

These data from simulations without any use of amylase in the oral phase corroborate previously published data showing ENZ’s proteolytic activity in typical human gastric acidity during oro-gastric simulations, albeit with the inappropriate use of porcine pancreatic amylase (PPA) in the oral phase as a replacement for human salivary amylase [24]. This distinction is important because PPA demonstrates unintended, robust proteolytic activity that may complicate the interpretation of protein hydrolysis by supplemental enzymes [47].

#### 3.2.2. Amino Acids

The average gastric digesta total AA concentrations were comparable between the standard and aging conditions (6.02 vs. 6.04 mg/g, *p* = 0.9204; Figure 2B and Table 2), suggesting that pepsin’s minor exopeptidase activity is not affected by the reduced gastric acidity in the aging condition. An interaction analysis did not reveal a condition-dependent effect on the increase in the total AAs with ENZ (*p* = 0.1345). In the standard condition, ENZ increased the total AA concentration by 18.3% compared to the control (7.12 vs. 6.02 mg/g, *p* = 0.0005; Figure 2B). In the aging condition, ENZ increased the total AA concentration by 25.9% compared to the control (7.60 vs. 6.04 mg/g, *p* < 0.0001; Figure 2B). ENZ’s exopeptidase activity with respect to total AA release was higher in the aging condition compared to the standard condition (7.60 vs. 7.12 mg/g, *p* = 0.0395; Figure 2B), suggesting again that ENZ mediates dietary protein hydrolysis and free AA release better with an increased duration of the gastric phase. Note that ENZ showed no difference in the total AA release, or the release of any AA category, at varying starting gastric pH values (Figure S2).

The sum of the nine EAAs needed in the diet (i.e., histidine, isoleucine, leucine, lysine, methionine, phenylalanine, threonine, tryptophan, and valine) was also measured. In control digestas, the average total EAA concentration was only slightly higher in the aging condition compared to the standard condition (1.38 vs. 1.25 mg/g, *p* = 0.0374; Figure 2C), again suggesting that pepsin’s minor exopeptidase activity is not meaningfully impacted by the reduced gastric acidity in the aging condition. In the standard condition, ENZ increased the total EAA concentration by 57.6% compared to the control (2.17 vs. 1.38 mg/g, *p* < 0.0001; Figure 2C). In the aging condition, ENZ increased the total EAA concentration by 100.4% compared to the control (2.50 vs. 1.25 mg/g, *p* < 0.0001; Figure 2C). It is again noteworthy that ENZ’s exopeptidase activity, in this case with respect to free EAA release, was higher in the aging vs. standard condition (2.50 vs. 2.17 mg/g, *p* = 0.0002; Figure 2C), further suggesting that ENZ’s proteolytic activity is enhanced with the greater duration of the gastric phase. The interaction between the treatment and condition was significant (*p* = 0.0002), reflecting the greater ENZ-mediated release of EAAs in the aging model. A similar pattern in the effects of treatment and condition were observed for the total BCAAs (Figure 2D and Table 2).

Intriguingly, ENZ appeared to have the greatest effect on generating free leucine compared to the aforementioned AA categories, highlighting ENZ’s leucine aminopeptidase activity. In the standard condition, ENZ increased the average gastric digesta leucine concentration by 249.9% compared to the control (0.44 vs. 0.13 mg/g, *p* < 0.0001; Figure 2E and Table 2). In the aging condition, ENZ increased the leucine concentration by 371.2% compared to the control (0.49 vs. 0.10 mg/g, *p* < 0.0001; Figure 2E). ENZ’s leucine aminopeptidase activity was higher in the aging condition compared to the standard condition (0.49 vs. 0.44 mg/g, *p* = 0.0029; Figure 2C), and the interaction between the treatment and condition was significant (*p* = 0.0018). These data show ENZ’s ability to function more effectively in the aging condition, further highlighting ENZ’s potential utility in improving protein digestion in older adults.

#### 3.2.3. Peptide Profiling by Size Exclusion Chromatography and SDS-PAGE

**Figure 3:**
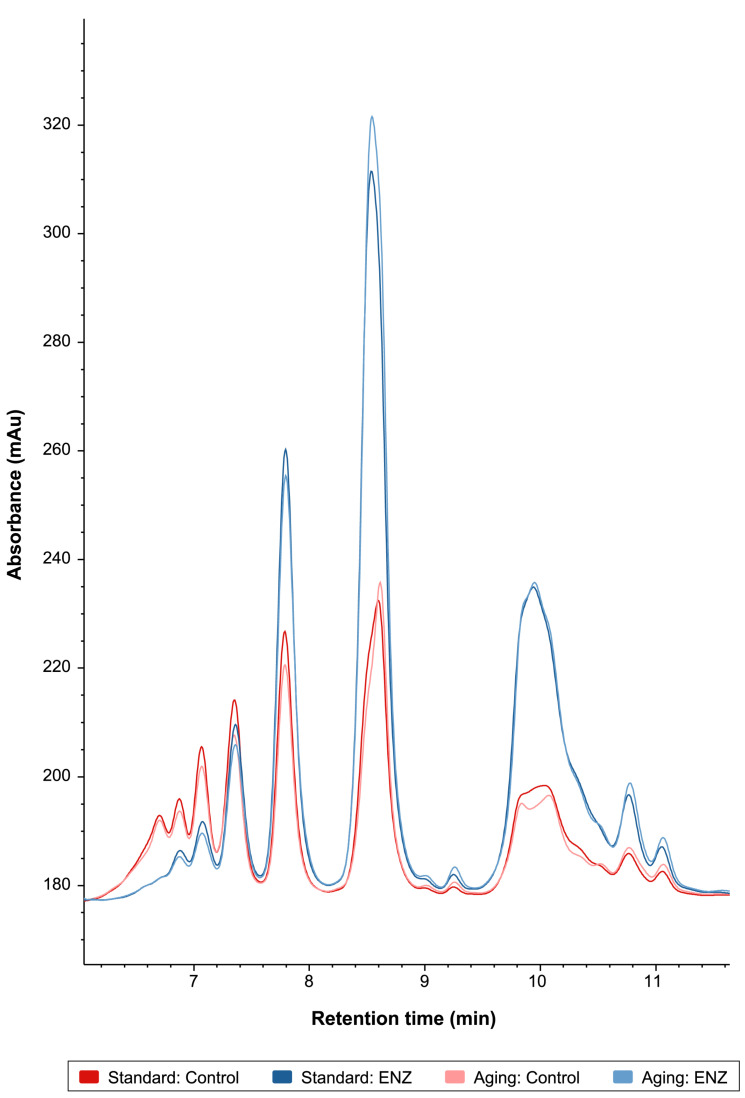
Size exclusion chromatography-based peptide distribution profiling of gastric digestas after oro-gastric simulations of mixed meal digestion with and without ENZ under standard INFOGEST 2.0 conditions (Standard) or conditions that model digestive decline with advancing age (Aging). The control simulations included porcine pepsin in the gastric phase. The ENZ simulations included porcine pepsin in the gastric phase along with a mixture of six microbial enzyme preparations. The retention times of t = 6.0 min and 7.5 min approximate peptides 20,000 and 5000 Da in size, respectively. The retention time of t = 11 min approximates free tryptophan standard (204 Da).

Gastric digestas were evaluated by size exclusion chromatography (SEC) to further evaluate protein digestion. Across all experimental groups, a total of 14 peaks were observed between retention times (RTs) t = 6.7 min and t = 11.1 min, corresponding to approximate peptide molecular weights (MWs) of 11,000 Da and 250 Da, respectively (Figure 3). Within all experimental groups, the peak with the highest absorbance occurred at t = 8.6 min, corresponding to approximately 1680 Da. Considering that the average MW of an AA is 110 Da, this maximum peak corresponds to a 15-AA long peptide. Within the control groups, the peptide distribution profiles showed similarities between the standard and aging conditions; however, the peak absorbances were lower in the aging condition for larger peptides over 3500 Da (i.e., the five peaks with the shortest RTs), reflecting the decreased pepsin concentration in the aging condition. ENZ use remarkably increased the release of shorter peptides less than 3500 Da, evidenced by increasing peak absorbances across the RT range of t = 7.8–11.1 min, compared to the pepsin-only control groups. The near absence of the first peak on the far left at t = 6.7 min in the ENZ groups, compared to the controls, and decreasing absorbances in the RT range of t = 6.8–7.4 min, reflect greater hydrolysis of larger peptides greater than 5500 Da than with pepsin alone.

**Figure 4:**
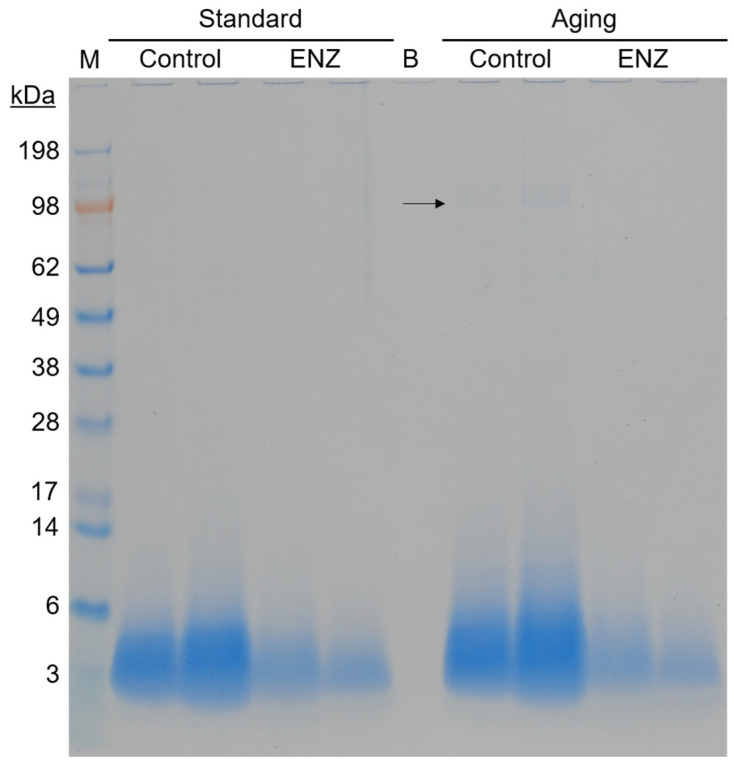
SDS-PAGE analysis of gastric digestas after oro-gastric simulations of mixed meal digestion with and without ENZ under standard INFOGEST 2.0 conditions (Standard) or conditions that model digestive decline with advancing age (Aging). The control simulations included porcine pepsin in the gastric phase. The ENZ simulations included porcine pepsin in the gastric phase along with a mixture of six microbial enzyme preparations. The arrow indicates a band that aligns with the molecular weight of pea lipoxygenase. Abbreviations: B—blank lane; M—molecular weight marker.

A confirmatory analysis using sodium dodecyl-sulfate polyacrylamide gel electrophoresis (SDS-PAGE) showed that the band smear intensity is visually decreased with ENZ use compared to the control in both conditions (Figure 4), especially for proteins ~3 kDa and larger, which corroborates the SEC data. Intriguingly, a lone pair of faint bands at ~98 kDa are only visible in the control digesta with the aging condition, suggesting that reduced pepsin usage or reduced acid-mediated hydrolysis led to the incomplete digestion of this protein. The MW of the band approximates the size of and matches the SDS-PAGE mobility profile of pea lipoxygenase [64], an abundant pea seed protein that is known to be highly stable and promote beany flavor notes [65]. Notably, the band is not visible in the aging condition with ENZ, suggesting that ENZ was capable of hydrolyzing pea lipoxygenase. Additional analyses using LC-MS peptide mapping were not conducted since the diversity of proteins in the mixed meal presents technical challenges to peptide identification. Future research with purer protein substrates (e.g., protein isolates) more amendable to SDS-PAGE and LC-MS analyses will be considered. Overall, the peptide distribution profiles by SEC and SDS-PAGE corroborate FAN and AA analyses, which suggest that ENZ mediates dietary protein hydrolysis better than pepsin alone.

The meaningfulness of proteolytic outcomes in this study was limited by the omission of an intestinal phase. Without an intestinal phase, it cannot be determined whether pancreatin, including pancreatic proteases, compensates for reduced gastric proteolysis in the aging condition. Pancreatin compensation was previously observed in a semi-dynamic model of aging using cooked lentils as a substrate [66]. Nonetheless, several static digestion simulation experiments with the INFOGEST aging protocol, and one similar protocol, suggested that protein hydrolysis remains reduced compared to the standard condition even after an intestinal phase with pancreatin is included [56,61,67]. It is also relevant to note that ENZ’s minor component protease—the protease from *Aspergillus melleus* (AmP)—was recently shown to improve protein digestibility (AmP: 63.6%; control: 55.6%) and peptide release in a semi-dynamic GI simulation of cooked bovine meat digestion under “elderly” conditions [27]. In this model, the elderly condition included 25% less pepsin, 50% less pancreatin, and a terminal gastric pH of 3 vs. pH 2 in the adult condition, amongst other condition-specific parameters [27]. Since the “adult” comparator in this study was only included as a control group without AmP, the differential effects of terminal gastric pH between adult and elderly conditions on AmP activity could not be evaluated [27]. AmP was studied at a dose of 15,000 hemoglobin unit tyrosine base (HUT), equivalent to 125 leucine aminopeptidase units (LAPUs), per 3.53 oz cooked beef. This HUT activity is at most 25% of the ENZ mixture, suggesting that ENZ would similarly improve protein digestibility in a full GI simulation. In relation to this, ENZ was shown to enhance amino acid bioaccessibility from a mixed meal in a semi-dynamic GI digestion simulation adapted to aging by 30% less porcine and pancreatin usage [68]. That study was limited, though, by the lack of a standard condition comparator and a comparison of ENZ vs. control without replication [68].

It remains unclear whether the amplitude of AA release by ENZ and relative increases with ENZ vs. pepsin-only controls in vitro (Figure 2) translate to changes in postprandial plasma aminoacidemia, increased skeletal muscle uptake of AAs, and increased muscle protein synthesis in vivo. Encouragingly, a mixture of three microbial protease preparations, including two of the proteases in the ENZ mixture, was shown to increase postprandial plasma net exposure to AAs compared to the placebo after the consumption of 25 g of pea protein isolate by healthy adults in a crossover clinical trial [36]. The ENZ mixture is currently under investigation in a similar plasma aminoacidemia trial in older adults with a mixed meal nearly identical to the mixed meal described in this study [69].

### 3.3. Fatty Acid and Glycerol Bioaccessibility

**Figure 5:**
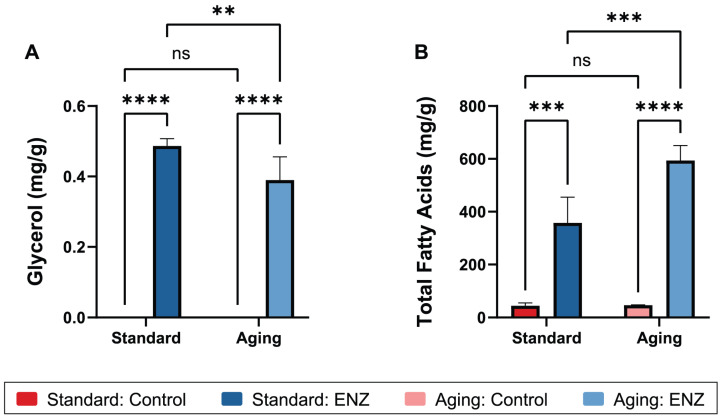
Average gastric digesta glycerol (**A**) and total fatty acid (**B**) concentrations after oro-gastric simulations of mixed meal digestion with and without ENZ under standard INFOGEST 2.0 conditions (Standard) or conditions that model digestive decline with advancing age (Aging). The control simulations included porcine pepsin in the gastric phase. The ENZ simulations included porcine pepsin in the gastric phase along with a mixture of six microbial enzyme preparations. The results are reported in mg analyte per g of mixed meal. The error bars show ± one standard deviation (*n* = 3 per group). **, *p* ≤ 0.01; ***, *p* ≤ 0.001, ****, *p* ≤ 0.0001; ns, not significant (*p* > 0.05).

FAs are released from triglycerides by the microbial lipase-mediated hydrolysis of all three fatty acid tails at the sn-1, sn-2, and sn-3 positions of the glycerol backbone [70]. Because rabbit gastric lipase was not included in this study, it is no surprise that the gastric digesta glycerol and total FA concentrations were not detectable and markedly lower, respectively, in the control groups compared to the ENZ-treated groups (Figure 5). ENZ use was associated with a higher average gastric digesta glycerol concentration compared to the controls (*p* < 0.0001). A significant interaction between treatment and condition was observed (*p* = 0.041), whereby ENZ use promoted a 22.2% higher glycerol concentration under the standard condition compared to the aging condition (0.44 vs. 0.36 mg/g, *p* = 0.0088; Figure 5A and Table 2).

ENZ use also resulted in a significant increase in the total FA concentrations of the oil layer of gastric digestas in both conditions compared to the controls (*p* < 0.0001; Figure 5B). In the standard condition, the total FA concentration increased from 44.5 mg/g in the control group to 357.7 mg/g with ENZ use (*p* = 0.0001). In the aging condition, ENZ use increased the total FA concentration from 46.5 mg/g in the control group to 593.5 mg/g (*p* < 0.0001). An interaction analysis revealed a significant interaction between the treatment and condition (*p* = 0.0073), whereby the increase in the total FA concentration with ENZ use in the aging condition was 65.9% higher compared to the standard condition (593.5 vs. 357.7 mg/g, *p* = 0.0009), suggesting enhanced free FA release in the aging condition.

There is an observed inconsistency between the glycerol and FA concentrations with ENZ use, whereby glycerol decreased and the total FA increased in the aging condition. One possible explanation is that the higher gastric pH of the aging condition reduced the complete hydrolysis of triglycerides, leading to stereospecific FA release and limited glycerol backbone release. Differences in the methods used to quantify glycerol and total FA may also have contributed to the observed discrepancy. Glycerol was measured in the whole supernatant of gastric digestas, while FAs were measured in the oil layer skimmed from the top of the supernatants. This difference in sampling could result in contrasting trends between the glycerol and FA concentrations under standard and aging conditions. Moreover, the glycerol signal intensity in the concentrated digesta was often below the limit of quantitation and close to the limit of detection. To ensure the measurement was within the quantifiable range, a glycerol standard equivalent to 0.1 mg/mL was added to the digesta samples, and this standard amount was subtracted from the final values. The high variance in the glycerol data, likely due to sample preparation and measurement methods, introduced additional uncertainty into these results. Glycerol measurement in future experimentation may be enhanced with the use of an evaporative light scattering detector, a solid-phase glycerol extraction method, measurement by gas chromatography, or higher starting loads of dietary fat in the mixed meal.

These glycerol and FA data demonstrate the acid-tolerant lipolytic activity of the *Candida cylindracea* lipase (CcL) in the ENZ mixture. Based on total FA release, CcL may show greater activity at a higher pH in the aging condition. This would be consistent with the known optimal pH range of CcL (Table 1) [71] and prior reports showing that secreted fungal lipases from *Candida* species function optimally at a neutral pH [72,73]. However, the observed inconsistency between glycerol and FA makes it difficult to draw reliable conclusions about the superior effectiveness of CcL in the aging condition compared to the standard condition. Furthermore, the lack of an experimental intestinal phase prevents any comparison of ENZ to pancreatic lipase and whether ENZ can compensate for reduced pancreatin usage in the aging model.

### 3.4. Maltose and Glucose Bioaccessibility

**Figure 6:**
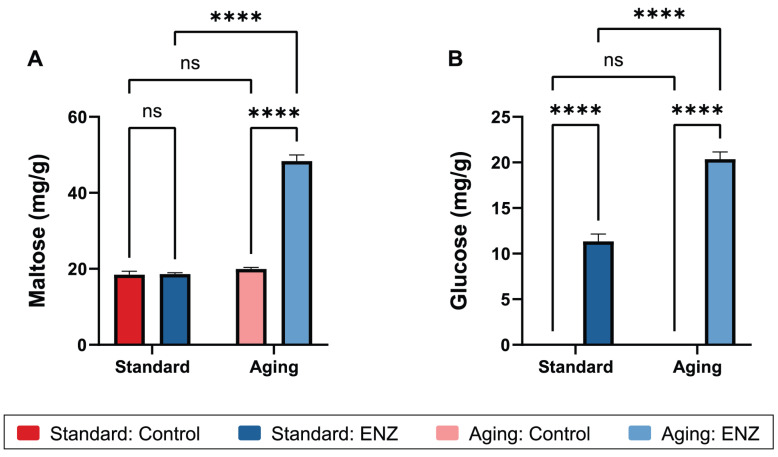
Average gastric digesta maltose (**A**) and glucose (**B**) concentrations after oro-gastric simulations of mixed meal digestion with and without ENZ under standard INFOGEST 2.0 conditions (Standard) or conditions that model digestive decline with advancing age (Aging). Control simulations included porcine pepsin in gastric phase. ENZ simulations included porcine pepsin in gastric phase along with mixture of six microbial enzyme preparations. Amylase was not included in oral phase. Results are reported in mg analyte per g of mixed meal. Error bars show ±1 standard deviation (*n* = 3 per group). ****, *p* ≤ 0.0001; ns, not significant (*p* > 0.05).

Maltose and glucose, indicators of starch digestion, were not expected to be generated under control conditions since amylase was optionally not used in the oral phase. Nonetheless, maltose was detected in control simulations, likely as a result of some acid hydrolysis of potato or pea starch in the mixed meal (Figure 6A and Table 2). Alternatively, these quantities of maltose may reflect the naturally occurring levels of maltose in the undigested meal at the start of the simulations. In the control groups, the gastric digesta maltose concentrations were comparable between the standard and aging conditions (18.5 vs. 20.0 mg/g, *p* = 0.089) (Figure 6A). Under the standard condition, the average maltose concentrations were comparable between the ENZ and control groups (18.5 vs. 18.6 mg/g, *p* = 0.87). In contrast, in the aging condition, the average maltose concentration increased by 142.1% with ENZ use compared to the control (48.4 vs. 20.0 mg/g, *p* < 0.0001; Figure 6A). The significant interaction between the treatment and condition (*p* < 0.0001) indicated that the effect of ENZ on the maltose concentration was condition-dependent, whereby the aging condition exhibited significantly enhanced maltose release. The greater amylolytic activity of ENZ in the aging model may reflect the pH-response profile of *Aspergillus oryzae* amylase, with optimal activity reported at approximately pH 5.0 (Table 1) [71]. Increased amylolysis in the aging condition may also be partly attributed to the extended duration of the gastric phase compared to the standard condition.

In the control groups, glucose was not detected in the gastric digesta in standard or aging conditions (Figure 6B), suggesting that the mixed meal contained very little free glucose prior to simulated digestion. This lack of glucose also suggests that the pea and potato starch in the meal are resistant to acid-mediated glucose release. The glucose concentrations increased with ENZ use in both the standard (*p* < 0.0001) and aging models (*p* < 0.0001) compared to the control groups. An interaction analysis revealed that the effect of ENZ on glucose release was significantly influenced by the condition (*p* < 0.0001), whereby the glucose concentration was 79.4% higher in the aging condition compared to the standard condition (20.1 vs. 11.2 mg/g, *p* < 0.0001). This glucose release is attributed to ENZ’s glucoamylase activity. Similarly to the *A. oryzae* amylase in the ENZ mixture, the *A. niger* glucoamylase functions optimally at approximately pH 4.5 (Table 1) [71]. Altogether, these in vitro digestion and pH response profile data suggest that ENZ’s glucoamylase and amylase activities may be higher in vivo under conditions of reduced gastric acidity, as has been observed in older adults compared to younger adults [5]. It is unclear what impact ENZ supplementation has on postprandial glycemia in vivo; however, improved glucose bioavailability could be beneficial for older adults who are malnourished or at risk for malnourishment. Ultimately, amylase and glucoamylase were included in the ENZ mixture to theoretically promote early gastric release of glucose to extend the postprandial duration of plasma glucose exposure and lower the amplitude of postprandial plasma glucose concentrations to promote metabolic and endocrine health. This theory is currently being tested in a clinical trial [69].

This study is limited with respect to carbohydrate digestion since neither human salivary amylase nor saliva was included in the oral phase. When either step was included, the gastric digesta glucose concentration or starch hydrolysis increased in the aging condition in studies on cooked lentils and lentil-based pasta [63,66] owing to human salivary amylase’s greater amylolytic activity at a neutral pH [74]. The lack of an experimental intestinal phase also prevents any comparison of ENZ to pancreatic amylase and whether ENZ can compensate for reduced pancreatin usage in the aging model.

## 4. Conclusions

Using the INFOGEST 2.0 in vitro static oro-gastric digestion simulation protocol, a mixture of six microbial enzyme preparations (ENZ) enhanced macronutrient digestion from a macronutrient-balanced mixed meal under both standard conditions and aging-adapted conditions defined by a consensus protocol. Analytical markers of protein, lipid, and carbohydrate hydrolysis were all increased in the aging condition compared to the standard condition, likely as a consequence of the greater duration of the gastric phase in the aging condition for the constituent protease preparations. The lipase, amylase, and glucoamylase components may also be assisted by greater enzyme activity at the slightly higher gastric pH used to model digestive senescence; however, additional research in dynamic digestion simulations with conditions that isolate pH as a lone variable is needed. Based on the preliminary findings reported herein, it is also recommended that no assumptions be made about the pH response of gastric pepsin or the generalizability of prior studies; pepsin activity’s pH response profile is likely substrate-dependent in gastric simulations, with emerging research supporting this characteristic [53]. This research builds on a prior study of the ENZ mixture in a preliminary non-consensus INFOGEST model of aging that confoundingly employed the use of porcine pancreatic amylase in the oral phase [24], which conferred unintended protease activity [47]. This research also aligns with a recent report showing that one of ENZ’s constituent proteases improves protein digestion from cooked beef in a dynamic gastrointestinal digestion simulation [27]. Altogether, these data help support the rationale for microbial enzyme supplementation with meals in older adults at risk for malnutrition and sarcopenia. The ENZ mixture is currently being evaluated in a clinical trial in older adults [69].

## Figures and Tables

**Table 1:** Composition of the supplemental mixture of six microbial enzyme preparations (ENZ) ^1^.

Primary Enzyme Activity	CAS No.	EC No.	Source Organism	Activity per Dose ^2^	Optimum pH (Range) ^3^
Protease	9025-49-4	3.4.23.18	*Aspergillus oryzae*	60,000 HUT	3.0 (3.0–6.0)
Protease	9025-49-4	3.4.23.18	*Aspergillus niger*	300 SAP	3.0 (1.5–4.5)
Protease	9074-07-1	3.4.21.63	*Aspergillus melleus*	50 LAPU	7.5 (5.5–10.0)
Lipase	9001-62-1	3.1.1.3	*Candida cylindracea*	3000 FIP	7.0 (3.0–9.0)
Amylase	9000-90-2	3.2.1.1	*Aspergillus oryzae*	10,000 SKB ^4^	5.0 (3.5–6.5)
Glucoamylase	9032-08-0	3.2.1.3	*Aspergillus niger*	25 AGU	4.5 (3.0–6.0)

^1^ Inactive ingredients include potato dextrin, tapioca maltodextrin, dextrose, calcium carbonate, potassium sorbate, sodium benzoate, and silicon dioxide. ^2^ Enzyme activity assays used to separately confirm the activity of each enzyme preparation were previously described [24]. The listed activity is specific to the individual enzyme preparation added to the mixture. The total activities of the ENZ mixture may be higher than listed due to the “side activities” of the individual enzyme preparations. For example, ENZ routinely shows proteolytic activity ≥ 80,000 HUT. ^3^ The values are according to the manufacturer or supplier’s technical documentation based on various assays that are amenable to pH adjustment. Bounds of the optimal pH range are defined by ≥50% optimal activity. ^4^ One SKB unit is equivalent to one dextrinizing unit (DU). Abbreviations: AGU—amyloglucosidase unit; CAS No.—Chemical Abstract Society Registry No.; EC No.—Enzyme Commission No.; FIP—Fédération Internationale Pharmaceutique unit; HUT—hemoglobin unit tyrosine base; LAPU—leucine aminopeptidase unit; SAP—spectrophotometric acid protease unit; SKB—Sandstedt, Kneen, and Blish method.

**Table 2:** The effects of a supplemental mixture of microbial enzymes on gastric digesta nutrient concentrations after simulated oro-gastric digestion of a mixed meal containing chicken meat, sweet peas, potatoes, butter, and milk.

Analyte (Units)	Gastric Digesta Concentration ^1^			*p* Values ^3^						
	Simulation Condition ^2^	Control	ENZ	*p_treatment_*	*p_condition_*	*p_interaction_*	Control vs. ENZ ^4^		Standard vs. Aging ^4^	
							Standard	Aging	Control	ENZ
FAN (mg N/g)	Standard	1.467 ± 0.021	2.043 ± 0.051	<0.0001	0.0002	<0.0001	<0.0001	<0.0001	<0.0001	0.6095
	Aging	1.163 ± 0.031	2.060 ± 0.044							
Total AA (mg/g)	Standard	6.020 ± 0.256	7.123 ± 0.210	<0.0001	0.1077	0.1345	0.0005	<0.0001	0.9204	0.0395
	Aging	6.040 ± 0.327	7.603 ± 0.081							
EAA (mg/g)	Standard	1.376 ± 0.041	2.169 ± 0.064	<0.0001	0.0188	0.0002	<0.0001	<0.0001	0.0374	0.0002
	Aging	1.250 ± 0.096	2.505 ± 0.016							
BCAA (mg/g)	Standard	0.412 ± 0.013	0.760 ± 0.028	<0.0001	<0.0001	<0.0001	<0.0001	<0.0001	0.0009	<0.0001
	Aging	0.525 ± 0.037	1.503 ± 0.024							
Leucine (mg/g)	Standard	0.127 ± 0.006	0.443 ± 0.021	<0.0001	0.206	0.0018	<0.0001	<0.0001	0.0528	0.0029
	Aging	0.103 ± 0.006	0.487 ± 0.012							
Glycerol (mg/g)	Standard	0	0.443 ± 0.255	<0.0001	0.0410	0.0410	<0.0001	<0.0001	>0.9999	0.0088
	Aging	0	0.357 ± 0.212							
Total FA (mg/g)	Standard	44.5 ± 10.4	357.7 ± 97.6	<0.0001	0.0067	0.0073	0.0001	<0.0001	0.9668	0.0009
	Aging	46.5 ± 0.9	593.5 ± 57.0							
Maltose (mg/g)	Standard	18.49 ± 0.893	18.62 ± 0.339	<0.0001	<0.0001	<0.0001	0.8677	<0.0001	0.0890	<0.0001
	Aging	19.99 ± 0.390	48.39 ± 1.593							
Glucose (mg/g)	Standard	0	11.20 ± 0.81	<0.0001	<0.0001	<0.0001	<0.0001	<0.0001	>0.9999	<0.0001
	Aging	0	20.09 ± 0.79							

^1^ The data are expressed as the means ± one standard deviation (*n* = 3). ^2^ The standard condition is defined by the consensus INFOGEST 2.0 static digestion simulation protocol [38]. The aging condition is defined by the consensus INFOGEST static digestion simulation protocol adapted to older adults [41]. ^3^ A two-way analysis of variance (ANOVA) was conducted to evaluate the effects of the treatment (control or ENZ), digestion simulation condition (standard or aging), and the interaction between the treatment and condition. ^4^ Following the ANOVA, the uncorrected Fisher’s least significant difference test was applied for pairwise comparisons within each treatment and condition. Abbreviations: AA—amino acid; BCAA—total branched chain amino acid; EAA—total essential amino acid; ENZ—mixture of six microbial enzyme preparations; FAN—free amino nitrogen.

## Data Availability

The raw data supporting the conclusions of this article will be made available by the authors upon request.

## Supplementary Materials

The following supporting information can be downloaded at https://www.mdpi.com/article/10.3390/foods14060937/s1, Figure S1: Preliminary pH-response profile of ENZ’s proteolytic activity according to a modified hemoglobin unit tyrosine base (HUT) assay.; Figure S2: Preliminary pH-response profiling of pepsin and ENZ in a modified INFOGEST 2.0 static simulation of oro-gastric digestion.

## Abbreviations

The following abbreviations are used in this manuscript:

AA	amino acid
AmP	*Aspergillus melleus* protease
ANOVA	analysis of variance
BCAA	branched chain amino acid
CcL	*Candida cylindracea* lipase
EAA	essential amino acid
ENZ	mixture of six microbial enzyme preparations
FA	fatty acid
FAN	free amino nitrogen
GI	gastrointestinal
MW	molecular weight
NAC	N-acetyl-L-cysteine
OPA	o-phthaldaldehyde
PPA	porcine pancreatic amylase
PVDF	polyvinylidenedifluoride
RT	retention time
SDS-PAGE	sodium dodecyl-sulfate polyacrylamide gel electrophoresis
SEC	size exclusion chromatography

## References

[1] Rémond D., Shahar D.R., Gille D., Pinto P., Kachal J., Peyron M.A., Dos Santos C.N., Walther B., Bordoni A., Dupont D. (2015). Understanding the gastrointestinal tract of the elderly to develop dietary solutions that prevent malnutrition. Oncotarget.

[2] Feldman M., Cryer B., McArthur K.E., Huet B.A., Lee E. (1996). Effects of aging and gastritis on gastric acid and pepsin secretion in humans: A prospective study. Gastroenterology.

[3] Feldman M., Cryer B. (1998). Effects of age on gastric alkaline and nonparietal fluid secretion in humans. Gerontology.

[4] Meyer J., Spier E., Neuwelt F. (1940). Basal secretion of digestive enzymes in old age. Arch. Intern. Med..

[5] Dreiling D.A., Triebling A.T., Koller M. (1985). The effect of age on human exocrine pancreatic secretion. Mt. Sinai J. Med..

[6] Russell T.L., Berardi R.R., Barnett J.L., Dermentzoglou L.C., Jarvenpaa K.M., Schmaltz S.P., Dressman J.B. (1993). Upper gastrointestinal pH in seventy-nine healthy, elderly, North American men and women. Pharm. Res..

[7] Clarkston W.K., Pantano M.M., Morley J.E., Horowitz M., Littlefield J.M., Burton F.R. (1997). Evidence for the anorexia of aging: Gastrointestinal transit and hunger in healthy elderly vs. young adults. Am. J. Physiol..

[8] Di Francesco V., Zamboni M., Dioli A., Zoico E., Mazzali G., Omizzolo F., Bissoli L., Solerte S.B., Benini L., Bosello O. (2005). Delayed postprandial gastric emptying and impaired gallbladder contraction together with elevated cholecystokinin and peptide YY serum levels sustain satiety and inhibit hunger in healthy elderly persons. J. Gerontol. A Biol. Sci. Med. Sci..

[9] O’Donovan D., Hausken T., Lei Y., Russo A., Keogh J., Horowitz M., Jones K.L. (2005). Effect of aging on transpyloric flow, gastric emptying, and intragastric distribution in healthy humans--impact on glycemia. Dig. Dis. Sci..

[10] Soenen S., Rayner C.K., Horowitz M., Jones K.L. (2015). Gastric emptying in the elderly. Clin. Geriatr. Med..

[11] Fikry M.E. (1968). Exocrine pancreatic functions in the aged. J. Am. Geriatr. Soc..

[12] Ishibashi T., Matsumoto S., Harada H., Ochi K., Tanaka J., Seno T., Oka H., Miyake H., Kimura I. (1991). Aging and Exocrine Pancreatic Function Evaluated by the Recently Standardized Secretin Test. Jpn. J. Geriatr..

[13] Laugier R., Bernard J.P., Berthezene P., Dupuy P. (1991). Changes in pancreatic exocrine secretion with age: Pancreatic exocrine secretion does decrease in the elderly. Digestion.

[14] Vellas B., Balas D., Moreau J., Bouisson M., Senegas-Balas F., Guidet M., Ribet A. (1988). Exocrine pancreatic secretion in the elderly. Int. J. Pancreatol..

[15] Keller J., Layer P. (2005). Human pancreatic exocrine response to nutrients in health and disease. Gut.

[16] Einarsson K., Nilsell K., Leijd B., Angelin B. (1985). Influence of age on secretion of cholesterol and synthesis of bile acids by the liver. N. Engl. J. Med..

[17] Salemans J.M., Nagengast F.M., Tangerman A., van Schaik A., Hopman W.P., de Haan A.F., Jansen J.B. (1993). Effect of ageing on postprandial conjugated and unconjugated serum bile acid levels in healthy subjects. Eur. J. Clin. Investig..

[18] Sanders L., Goltz S., Maki K.C. (2023). Resiliency of the digestive system during aging and the impact of diet. Nutr. Today.

[19] Morley J.E. (1997). Anorexia of aging: Physiologic and pathologic. Am. J. Clin. Nutr..

[20] Morley J.E., Silver A.J., Miller D.K., Rubenstein L.Z. (1989). The anorexia of the elderly. Ann. N. Y. Acad. Sci..

[21] Calvani R., Picca A., Coelho-Júnior H.J., Tosato M., Marzetti E., Landi F. (2023). Diet for the prevention and management of sarcopenia. Metabolism.

[22] Morley J.E., Argiles J.M., Evans W.J., Bhasin S., Cella D., Deutz N.E., Doehner W., Fearon K.C., Ferrucci L., Hellerstein M. (2010). Nutritional recommendations for the management of sarcopenia. J. Am. Med. Dir. Assoc..

[23] Robinson S., Granic A., Cruz-Jentoft A.J., Sayer A.A. (2023). The role of nutrition in the prevention of sarcopenia. Am. J. Clin. Nutr..

[24] Garvey S.M., Guice J.L., Hollins M.D., Best C.H., Tinker K.M. (2022). Fungal digestive enzymes promote macronutrient hydrolysis in the INFOGEST static in vitro simulation of digestion. Food Chem..

[25] Freitas D., Gómez-Mascaraque L.G., Brodkorb A. (2022). Digestion of protein and toxic gluten peptides in wheat bread, pasta and cereal and the effect of a supplemental enzyme mix. Front. Nutr..

[26] Tran Do D.H., Kong F., Penet C., Winetzky D., Gregory K. (2016). Using a dynamic stomach model to study efficacy of supplemental enzymes during simulated digestion. LWT–Food Sci. Technol..

[27] Zhou Z., Liu Y., Ishigaki Y., Yamaguchi S., Chen J., Liu X. (2025). Microbial protease supplementation improves gastric emptying and protein digestive fate of beef for the elderly under dynamic in vitro digestion. Food Res. Int..

[28] Menden A., Hall D., Broedlow C.A., Darcey T., Crawford F., Klatt N., Crynen S., Mullan M., Ait-Ghezala G. (2020). *Candida rugosa* lipase alters the gastrointestinal environment in wild-type mice. Biomed. Pharmacother..

[29] Menden A., Hall D., Hahn-Townsend C., Broedlow C.A., Joshi U., Pearson A., Crawford F., Evans J.E., Klatt N., Crynen S. (2022). Exogenous lipase administration alters gut microbiota composition and ameliorates Alzheimer’s disease-like pathology in APP/PS1 mice. Sci. Rep..

[30] Liang Q., Yuan M., Xu L., Lio E., Zhang F., Mou H., Secundo F. (2022). Application of enzymes as a feed additive in aquaculture. Mar. Life Sci.Technol..

[31] Torres-Pitarch A., Manzanilla E.G., Gardiner G.E., O’Doherty J.V., Lawlor P.G. (2019). Systematic review and meta-analysis of the effect of feed enzymes on growth and nutrient digestibility in grow-finisher pigs: Effect of enzyme type and cereal source. Anim. Feed Sci. Technol..

[32] Ido H., Matsubara H., Kuroda M., Takahashi A., Kojima Y., Koikeda S., Sasaki M. (2018). Combination of gluten-digesting enzymes improved symptoms of non-celiac gluten sensitivity: A randomized single-blind, placebo-controlled crossover study. Clin. Transl. Gastroenterol..

[33] Majeed M., Majeed S., Nagabhushanam K., Arumugam S., Pande A., Paschapur M., Ali F. (2018). Evaluation of the safety and efficacy of a multienzyme complex in patients with functional dyspepsia: A randomized, double-blind, placebo-controlled study. J. Med. Food.

[34] Martin-Biggers J. (2024). A multi-digestive enzyme and herbal dietary supplement reduces bloating in a single use in healthy adults: A randomized, placebo-controlled, cross over study. Nutr. Diet. Suppl..

[35] König J., Holster S., Bruins M.J., Brummer R.J. (2017). Randomized clinical trial: Effective gluten degradation by *Aspergillus niger*-derived enzyme in a complex meal setting. Sci. Rep..

[36] Paulussen K.J.M., Askow A.T., Deutz M.T., McKenna C.F., Garvey S.M., Guice J.L., Kesler R.M., Barnes T.M., Tinker K.M., Paluska S.A. (2024). Acute microbial protease supplementation increases net postprandial plasma amino acid concentrations after pea protein ingestion in healthy adults: A randomized, double-blind, placebo-controlled trial. J. Nutr..

[37] Minekus M., Alminger M., Alvito P., Ballance S., Bohn T., Bourlieu C., Carrière F., Boutrou R., Corredig M., Dupont D. (2014). A standardised static in vitro digestion method suitable for food—An international consensus. Food Funct..

[38] Brodkorb A., Egger L., Alminger M., Alvito P., Assunção R., Ballance S., Bohn T., Bourlieu-Lacanal C., Boutrou R., Carrière F. (2019). INFOGEST static in vitro simulation of gastrointestinal food digestion. Nat. Protoc..

[39] Mulet-Cabero A.I., Egger L., Portmann R., Ménard O., Marze S., Minekus M., Le Feunteun S., Sarkar A., Grundy M.M., Carrière F. (2020). A standardised semi-dynamic in vitro digestion method suitable for food—An international consensus. Food Funct..

[40] Egger L., Schlegel P., Baumann C., Stoffers H., Guggisberg D., Brügger C., Dürr D., Stoll P., Vergères G., Portmann R. (2017). Physiological comparability of the harmonized INFOGEST in vitro digestion method to in vivo pig digestion. Food Res. Int..

[41] Menard O., Lesmes U., Shani-Levi C.S., Araiza Calahorra A., Lavoisier A., Morzel M., Rieder A., Feron G., Nebbia S., Mashiah L. (2023). Static in vitro digestion model adapted to the general older adult population: An INFOGEST international consensus. Food Funct..

[42] Otten J.J., Pitzi Hellwig J., Meyers L.D., Institute of Medicine (2006). Chapter: Macronutrients, Healthful Diets, and Physical Activity. Dietary Reference Intakes: The Essential Guide to Nutrient Requirements.

[43] Campbell W.W., Deutz N.E.P., Volpi E., Apovian C.M. (2023). Nutritional interventions: Dietary protein needs and influences on skeletal muscle of older adults. J. Gerontol. A Biol. Sci. Med. Sci..

[44] Chiwele I., Jones B.E., Podczeck F. (2000). The shell dissolution of various empty hard capsules. Chem. Pharm. Bull..

[45] Medina Hernández M.J., Villanueva Camañas R.M., Monfort Cuenca E., García Alvarez-Coque M.C. (1990). Determination of the protein and free amino acid content in a sample using o-phthalaldehyde and N-acetyl-L-cysteine. Analyst.

[46] Rieder A., Afseth N.K., Böcker U., Knutsen S.H., Kirkhus B., Mæhre H.K., Ballance S., Wubshet S.G. (2021). Improved estimation of in vitro protein digestibility of different foods using size exclusion chromatography. Food Chem..

[47] Qu Y., Tinker K.M., Madden E.N., Best C.H., Farmar J.G., Garvey S.M. (2025). Comparative evaluation of amylases in the oral phase of the INFOGEST static simulation of oro-gastric digestion. Food Res. Int..

[48] Guarrasi V., Mangione M.R., Sanfratello V., Martorana V., Bulone D. (2010). Quantification of underivatized fatty acids from vegetable oils by HPLC with UV detection. J. Chromatogr. Sci..

[49] Jensen R.G. (2002). The composition of bovine milk lipids: January 1995 to December 2000. J. Dairy Sci..

[50] Brooks F.P. (1985). Effect of diet on gastric secretion. Amer. J. Clin. Nutr..

[51] Mennah-Govela Y.A., Cai H., Chu J., Kim K., Maborang M.K., Sun W., Bornhorst G.M. (2020). Buffering capacity of commercially available foods is influenced by composition and initial properties in the context of gastric digestion. Food Funct..

[52] Piper D.W., Fenton B.H. (1965). pH stability and activity curves of pepsin with special reference to their clinical importance. Gut.

[53] Salelles L., Floury J., Le Feunteun S. (2021). Pepsin activity as a function of pH and digestion time on caseins and egg white proteins under static in vitro conditions. Food Funct..

[54] Taylor W.H. (1959). Studies on gastric proteolysis. I. The proteolytic activity of human gastric juice and pig and calf gastric mucosal extracts below pH5. Biochem. J..

[55] Deng R., Nau F., Lucas T., Maillard M., Leduc A., Ossemond J., Musse M., Le Feunteun S. (2025). First assessments of nutrient bioaccessibility with an INFOGEST semi-dynamic gastric digestion in vitro protocol adapted to model proton pump inhibitor use. Food Res. Int..

[56] Sánchez-García J., Muñoz-Pina S., García-Hernández J., Tárrega A., Heredia A., Andrés A. (2024). Protein digestibility and ACE inhibitory activity of fermented flours in older adults and standard gastrointestinal simulation. Food Res. Int..

[57] Lavoisier A., Morzel M., Chevalier S., Henry G., Jardin J., Harel-Oger M., Garric G., Dupont D. (2023). In vitro digestion of two protein-rich dairy products in the ageing gastrointestinal tract. Food Funct..

[58] Lavoisier A., Chevalier S., Henry G., Ossemond J., Harel-Oger M., Garric G., Dupont D., Morzel M. (2024). Impact of age on the digestion of cream cheese formulated with opposite caseins to whey proteins ratios: An in vitro study. Food Res. Int..

[59] Wang Z., Liu D., Hong X., Tao X., Zhang J., Zhang J., Hou Y., Wu T., Liu X., Zhou P. (2024). Calcium binding affects in vitro gastrointestinal digestion of bovine α-lactalbumin under infant, adult and elderly conditions. Int. Dairy J..

[60] Cao X., Zhao F., Lin Z., Sun X., Zeng X., Liu H., Li Y., Yuan Z., Su Y., Wang C. (2024). In vitro digestion mimicking conditions in adults and elderly reveals digestive characteristics of pork from different cooking ways. Food Res. Int..

[61] Qiu Z., Shi Y., Zheng Y., Shi W., Zhang L., Yin M., Wang X. (2025). Comparison of in vitro digestive characteristics of proteins from different sources in simulated elderly gastrointestinal conditions. Food Chem..

[62] Ribes S., Arnal M., Talens P. (2023). Influence of food oral processing, bolus characteristics, and digestive conditions on the protein digestibility of turkey cold meat and fresh cheese. Food Res. Int..

[63] Arnal M., Salcedo L., Talens P., Ribes S. (2024). Role of food texture, oral processing responses, bolus properties, and digestive conditions on the nutrient bioaccessibility of al dente and soft-cooked red lentil pasta. Foods.

[64] García Arteaga V., Kraus S., Schott M., Muranyi I., Schweiggert-Weisz U., Eisner P. (2021). Screening of twelve pea (*Pisum sativum* L.) cultivars and their isolates focusing on the protein characterization, functionality, and sensory profiles. Foods.

[65] Lao Y., Ye Q., Wang Y., Selomulya C. (2024). Modulating digestibility and mitigating beany flavor of pea protein. Food Rev. Int..

[66] Duijsens D., Verkempinck S.H.E., Somers E., Hendrickx M.E.G., Grauwet T. (2024). From static to semi-dynamic in vitro digestion conditions relevant for the older population: Starch and protein digestion of cooked lentils. Food Funct..

[67] Hernández-Olivas E., Muñoz-Pina S., Andrés A., Heredia A. (2020). Impact of elderly gastrointestinal alterations on in vitro digestion of salmon, sardine, sea bass and hake: Proteolysis, lipolysis and bioaccessibility of calcium and vitamins. Food Chem..

[68] Garvey S., Lange K., Van Den Ende T., Tinker K. Microbial enzymes enhance macronutrient digestibility in a dynamic digestion simulation. Proceedings of the 8th International Conference on Food Digestion.

[69] ClinicalTrials.gov. https://clinicaltrials.gov/study/NCT05211440.

[70] Chandra P., Enespa, Singh R., Arora P.K. (2020). Microbial lipases and their industrial applications: A comprehensive review. Microb. Cell Fact..

[71] Garvey S.M., Tinker K.M., Hollins M.D., Guice J.L., Schuler C. (2024). Fungal Enzyme Mixtures and Uses Thereof. U.S. Patent.

[72] Domínguez de María P., Sánchez-Montero J.M., Sinisterra J.V., Alcántara A.R. (2006). Understanding *Candida rugosa* lipases: An overview. Biotechnol. Adv..

[73] Yeşiloğlu Y., Şit L. (2011). Biochemical properties of free and immobilized *Candida rugosa* lipase onto Al_2_O_3_: A comparative study. Artif. Cells Blood Substitut. Immobil. Biotechnol..

[74] Freitas D., Le Feunteun S., Panouillé M., Souchon I. (2018). The important role of salivary α-amylase in the gastric digestion of wheat bread starch. Food Funct..

