# Supplementary material for: The Effects of a Microbial Enzyme Mixture on Macronutrient Hydrolysis in a Static Simulation of Oro-Gastric Digestion That Models Human Digestive Senescence
Source: Foods. 2025 Mar 10;14(6):937. doi: 10.3390/foods14060937 (PMC11941177; doi:10.3390/foods14060937)
Supplement: Supplementary file 1 [file foods-14-00937-s001.zip › foods-3457165-supplementary.pdf]

foods

MDPI

Supplementary Material

Supplementary Methods

pH-Response Profiling: Modified HUT Assay

The effect of pH on the proteolytic activity of a mixture of six microbial enzyme preparations (ENZ) was evaluated with a modified hemoglobin unit on tyrosine basis (HUT) assay. The standard Food Chemicals Codex HUT assay protocol is available online (https://www.foodchemicalscodex.org/). The standard HUT assay was adjusted to a smaller scale and modified to characterize HUT activity across a range of pH by preparing 12 different acetate buffers with a pH range of 2–6 in 0.5 unit increments and also pH 3.7, which is the starting gastric pH of the INFOGEST 2.0 static digestion simulation adapted to older adults. Each buffer was adjusted using 2 M sodium acetate or 1 M hydrochloric acid and used to prepare solutions of ENZ. The hemoglobin substrate solutions were prepared by dissolving 1 g hemoglobin from bovine blood (Product No. H2625, MilliporeSigma) in 25 mL water, stirring for 10 min, then adjusting pH to 1.7 using 1 M hydrochloric acid, with constant stirring for an additional 10 min. Samples were adjusted to the desired pH using 2 M sodium acetate or 1 M hydrochloric acid. Each substrate solution was then diluted to 50 mL with deionized water. A trichloroacetic acid (TCA) solution was prepared by dissolving 14 g TCA in water and diluting to 100 mL.

To begin the assay, 0.5 mL hemoglobin solution was pipetted into 2 mL microcentrifuge tubes: (i) two per enzyme sample, (ii) one for an enzyme blank, and (iii) one for a substrate blank. Test tubes were placed in a 40°C water bath to equilibrate for 5 min. 0.1 mL ENZ was pipetted into each tube containing hemoglobin and gently mixed. 0.1 mL acetate buffer was added to substrate blanks instead of ENZ. After a 30 min incubation, 0.5 mL TCA solution was added to each tube to stop the reaction. For the enzyme blank, 0.5 mL substrate and 0.5 mL TCA were combined and shaken gently, then 0.1 mL ENZ was added. Tubes were vortexed, left to cool to room temperature, and centrifuged at 10,000 rpm for 10 min. Absorbance of each supernatant was determined in a 1-cm quartz cuvette at 275 nm. One HUT unit of proteolytic activity is defined as the amount of enzyme that, in 1 min under the specified conditions, produces a hydrolysate whose absorbance at 275 nm is the same as that of a solution containing 1.10 ug per mL tyrosine in 0.006 N hydrochloric acid.

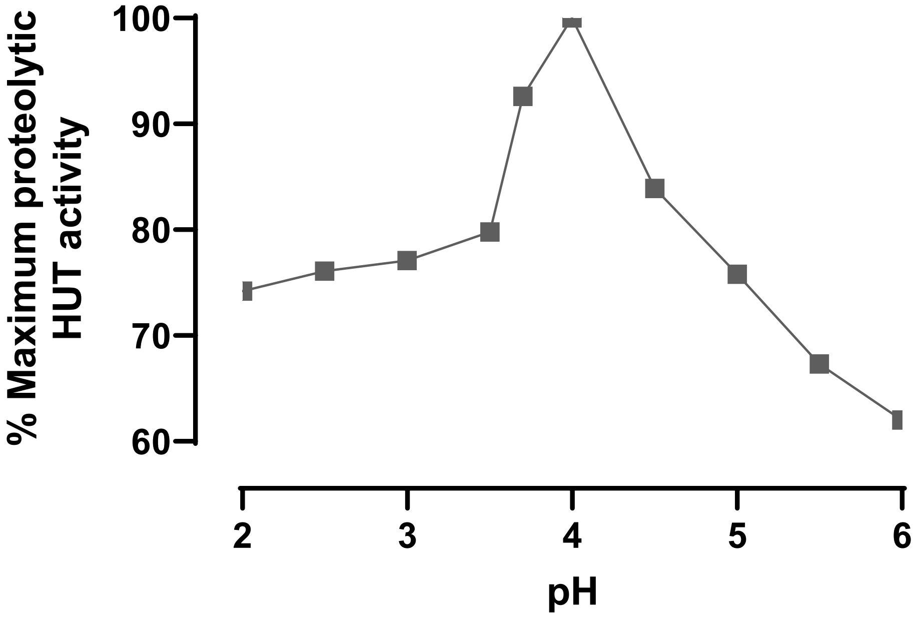

Figure S1. Preliminary pH-response profile of ENZ's proteolytic activity according to a modified hemoglobin unit tyrosine base (HUT) assay. The Food Chemicals Codex HUT assay is routinely performed at pH 4.7. In this modified assay, pH was adjusted to pH 2–6 in increments of 0.5 units, as well as pH 3.7, which is the starting gastric pH of the INFOGEST 2.0 protocol adapted to the study of older adults. Spectrophotometric analysis was carried out in duplicate with samples from only a single experiment at each pH (n = 1). Abbreviation: ENZ — mixture of six microbial enzyme preparations.

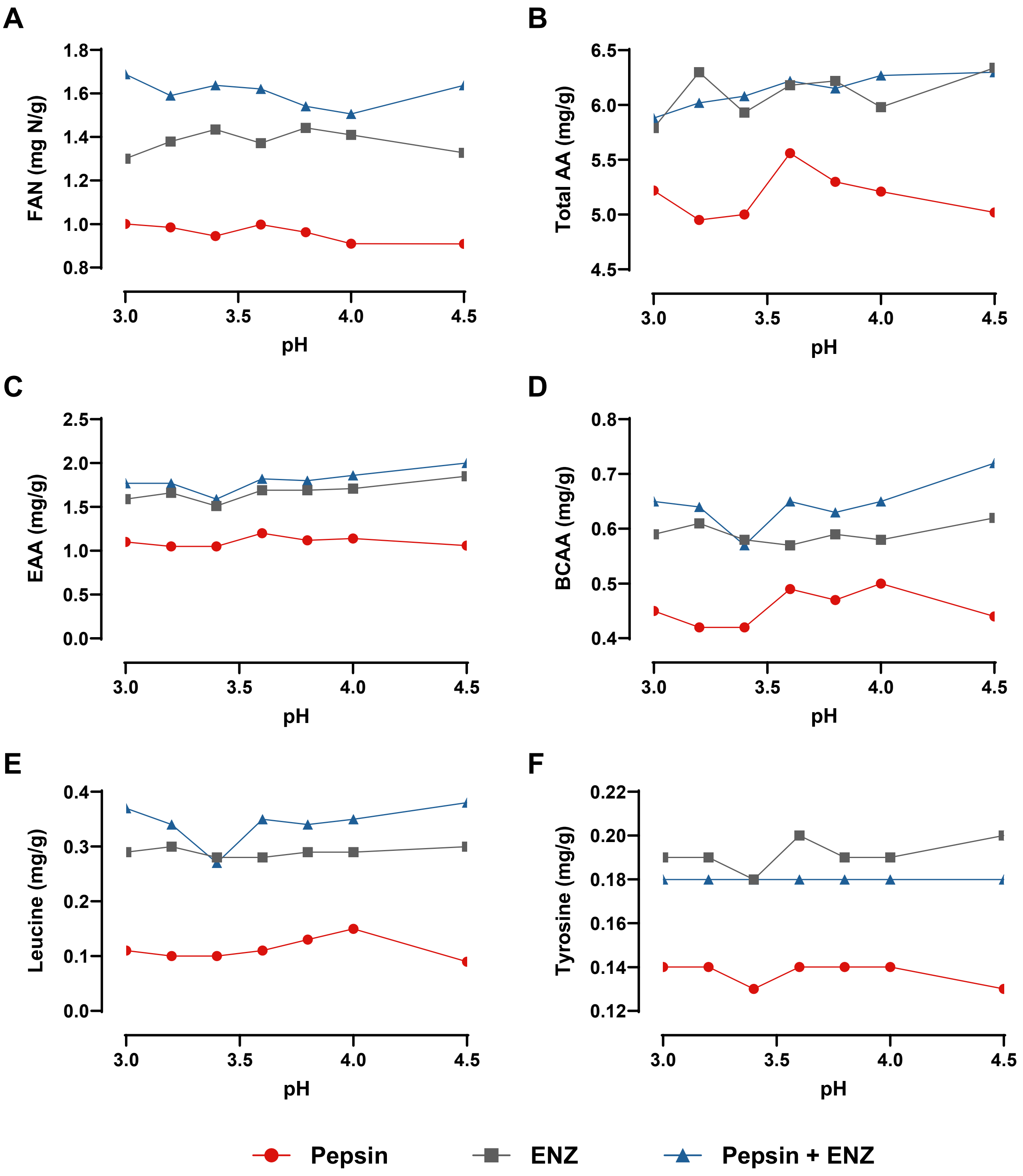

Figure S2. Preliminary pH-response profiling of pepsin and ENZ in a modified INFOGEST 2.0 static simulation of oro-gastric digestion. Shown are gastric digesta free amino nitrogen (FAN) (**A**), total amino acid (AA) (**B**), total essential amino acid (EAA) (**C**), total branched chain amino acid (BCAA) (**D**), leucine (**E**), and tyrosine (**F**) concentrations after oro-gastric simulation of mixed meal digestion with pepsin, ENZ, and pepsin with ENZ, under standard INFOGEST 2.0 conditions with a range of starting gastric pH and standard 2 h gastric phase duration. Results are reported in mg N or mg AA per g mixed meal. HPLC analysis was carried out with a single digesta sample from only a single simulation at each pH (n = 1). Abbreviation: ENZ — mixture of six microbial enzyme preparations.
